# Stride and Step Length Obtained with Inertial Measurement Units during Maximal Sprint Acceleration

**DOI:** 10.3390/sports7090202

**Published:** 2019-08-31

**Authors:** Cornelis J. de Ruiter, Jaap H. van Dieën

**Affiliations:** Department of Human Movement Sciences, Faculty of Behavioral and Movement Sciences, Vrije Universiteit Amsterdam, Amsterdam Movement Sciences, Van der Boechorststraat 9, 1081 BT Amsterdam, The Netherlands

**Keywords:** IMU, velocity, athletics, sprint start

## Abstract

During sprint acceleration, step length, step rate, ground contact, and airtime are key variables for coaches to guide the training process and technical development of their athletes. In the field, three of these variables are easily obtained with inertial measurement units (IMUs), but, unfortunately, valid estimates of step length with IMUs currently are limited to low speeds (<50% max). A simple method is proposed here to derive step length during maximal sprint acceleration, using IMUs on both feet and two timing gates only. Mono-exponential velocity-time functions are fitted to the 30-m (split) and 60-m times, which in combination with IMU-derived step durations yield estimates of step length. To validate this approach, sixteen well-trained athletes with IMUs on the insteps of both feet executed two 60-m maximal sprints, starting from a three-point position. As a reference, step lengths were determined from video data. The reference step lengths combined with IMU-derived step durations yielded a time series of step velocity that confirmed the appropriateness of a mono-exponential increase of step velocity (R^2^ ≥ 0.96). The comparison of estimated step lengths to reference measurements showed no significant difference (*p* > 0.05) and acceptable agreement (root mean square error, RMSE = 8.0 cm, bias ± Limits of Agreement = −0.15 ± 16 cm). Step length estimations further improved (RMSE = 5.7 cm, −0.16 ± 11 cm) after smoothing the original estimated step lengths with a third order polynomial function (R^2^ = 0.94 ± 0.04). In conclusion, during maximal sprint acceleration, acceptable estimates of stride and step length were obtained from IMU-derived step times and 30-m (split) and 60-m sprint times.

## 1. Introduction

In many sports, sprint acceleration is essential for good performance. Sprint speed is the product of step rate and step length [1]. Therefore, these variables, together with the ground contact time and the time between ground contacts (airtime), are frequently measured [2,3,4,5,6,7,8] and used by coaches to guide the training process and technical development of their athletes. Step rate, ground contact time, and airtime are readily obtained with inertial measurement units (IMUs) that measure acceleration, angular velocity, and the magnetic field in three orthogonal axes. IMUs are small, light-weight, inexpensive and are easily attached to the shoes of the athletes [9,10], making them ideal for use in sports practice on a day to day basis. Unfortunately, there are methodological challenges that prohibit accurate step length determination from IMU-signals [11,12]. 

Optoelectronic systems are the gold standard for motion analysis in sports and rehabilitation [13,14] and can even be used to accurately analyze fine motor skills [15,16]. However, optical motion capture systems are expensive, not easy to operate, and have some important practical limitations that hamper everyday use outside the laboratory. Multiple cameras are involved and a large number of markers have to be placed that often are difficult to track during complex sport motions. Wearable technology may overcome many of these practical limitations. IMUs in particular, may serve as an easily applicable, low cost alternative [13]. With IMUs, joint kinematics during walking and slow running can be obtained with reasonable accuracy (RMSE 3–7° during running) [17], but estimates of ground reaction forces and joint moments are still considerably less accurate than measurements done with optoelectronic systems and force platforms [14]. 

The present study focuses on step length during maximal sprint running. In a recent study, step length (and joint kinematics) during sprinting was recorded with an optoelectronic set up consisting of 21 cameras [18]. Fortunately, there are simpler optical measurement systems to obtain step length reliably [2], these are integrated into bars that can placed on both sides along the running lane [2,7] However, and similar to the more advanced motion capture systems, these simpler systems also are heavy, relatively expensive, and still difficult to use on a day to day basis at different locations. Consequently, in sports practice, step length during maximal sprint (acceleration) is often obtained with video, which is easy to use and less expensive than the other optical methods. However, video analysis is time consuming and the estimated step lengths may have limited accuracy, especially when the camera is not placed exactly perpendicular to the plane of progression of the runner, which often is the case. 

From a practical perspective, recording step length with IMUs worn on the feet is very attractive. Unfortunately, as indicated before, step length cannot be directly obtained from the IMU signals. With current sensor fusion algorithms that use the IMU signals as input, reasonably accurate estimations of stride length (a complete cycle of two consecutive steps) can only be obtained for running at slow (4–5 m·s^−1^) and constant velocities [11,12]. In contrast, the present study proposes a simple method to estimate step length during maximal velocity sprints including the acceleration phase. In this method, IMUs are attached to the athlete’s feet and to two mechanical timing gates. The use of mechanical timing gates equipped with IMUs in the present study guarantees that the 30-m and 60-m sprint times are carefully synchronized with the step times for all athletes. It is shown that mono-exponential velocity-time functions, solely based on the 30-m (split) and 60-m sprint times, appropriately describe the increase of step velocity during maximal sprint accelerations. Subsequently, it is demonstrated that these step velocity-time functions, in combination with IMU-derived step durations, yield acceptable estimates of step (and stride) length. The charm of the proposed step-length estimation method is that it is much cheaper and simpler to use in daily practice than currently available optical systems. Moreover, IMUs already provide accurate estimations of stride rate, ground contact, and airtimes, which in addition to step length, are key variables for coaches striving to enhance sprint acceleration of their athletes. 

During maximal sprint acceleration, the athlete’s (approximated) center of mass-velocity rapidly increases after the start. Following six to eight steps the velocity increase becomes more gradual until maximal velocity is reached around thirty meters into the sprint. Mathematically, such a velocity-time curve can be described by a mono-exponential function [3,19]. The first hypothesis of the present study was, that similar to what has been reported for the center of mass, individual mono-exponential functions would also accurately describe the athletes’ step to step velocity increase during maximal sprint acceleration. 

The second hypothesis was that two timing-gates would suffice to obtain these individual mono-exponential step-velocity-time functions. To this end, the timing-gates were strategically placed at thirty and sixty meters, to cover both the acceleration and the maximal velocity phase of sprinting. 

The third and main hypothesis was that concurrent validity of the step (and stride) lengths estimated with the IMUs compared to video-derived reference values would be good.

## 2. Materials and Methods

### 2.1. Subjects

Sixteen track and field athletes (3 females and 13 males) participated in this study. They were informed of the benefits and risks of the investigation prior to providing written informed consent. Their age, height, and body mass (means ± SD), respectively, were: 20.0 ± 3.1 year, 1.83 ± 0.08 m and 74.1 ± 9.2 kg. The study was conducted according to the declaration of Helsinki and approved by the local ethics committee (VCWE-2016-181R1). Most participants trained specifically for sprint events, but one female was a heptathlon athlete and among the males there was a high jumper, a triple jumper, a javelin thrower, and a middle distance runner. Thirteen athletes competed at the national (senior or junior) level and they all trained 4 to 6 times per week. Their 100-m personal best time was 12.10 ± 0.97 s (range: 10.50–13.90 s). All participants performed sprint exercises as part of their regular training schedule.

### 2.2. Test Procedures

The measurements took place in an indoor facility with a tartan floor at an ambient temperature of 20 °C. The participants did their preferred warm-up that was similar to what they would do when preparing for a competition. This included maximal acceleration runs performed on spiked shoes towards the end of the warm-up. Subsequently, custom-made (35 × 25 × 10 mm, 11 g) 3D IMUs (MPU-9150, Invensense, San Jose, CA, USA) were taped onto the instep of the shoes of all participants. The custom-made IMUs consisted of a gyroscope, with a range of +/− 2000°·s^−1^, accelerometers with a range of +/− 16 g, and magnetometers, which were switched off to save battery power (rechargeable lithium ion polymer 3.7 V, ICP 501421PS-01, Renata SA, Itingen, Switzerland). The data were logged onto an SD-card at a sample frequency of 500 Hz and were uploaded via USB after the measurements. 

In principle, the presented method to estimate step length will also work with optical timing gates. However, in the present study, custom-made mechanical timing gates equipped with IMUs were used. These have some practical advantages over optical systems, since these do not require access to electricity sockets or any cables. Moreover, the mechanical timing gates can easily be synchronized with the IMUs attached to the shoes of the athletes (see below). For time registration, an IMU was strapped to two lightweight rods made of carbon fiber covered with foam (1.5 m length and 1.5 cm cross-section, including the foam). Each rod was put into a custom-made pivot that was mounted on top of a tripod, which was placed next to the running lane. The rods were placed at chest height above the running lane at 30 m and 60 m from the start line. These rods easily rotated away when hit by a passing runner and subsequently returned to the neutral position by a set of small springs integrated in the pivot. The impact of a passing runner with the rod was registered by the IMUs (see data analysis). The mechanical timing gates were validated (y = 1.011x − 0.063, r^2^ = 0.999) against optical timing gates in a separate (unpublished) study with 16 other participants who ran flying sprints of 30 m at speeds that ranged from 3.5 to 10.0 m·s^−1^. 

Before the sprint tests, all IMUs were tightly packed together in a small box, and each of them was switched on manually. The box was closed by a lid preventing any movement of the IMUs relative to the box. Subsequently, a thud with the box on a hard surface caused a synchronized spike in the acceleration signals of all IMUs. The onset of these spikes was used to time-synchronize all IMUs during data analysis. Thereafter, when the athletes had completed their warm-up, the IMUs were strapped to their shoes and to both timing rods, and continued to sample for the entire duration of the test. 

The athletes started from what is known as a three-point position: With one hand on the floor, their front foot placed exactly against the start line and with their rear foot about one foot further backwards. The rear foot is the first to leave the ground during a sprint start. At the beginning of the push-off, there is a slight dorsal flexion in both ankles, which results in a negative time derivative of the angular velocity (unfiltered) around the x-axis of the IMUs. The last moment before the start of the push-off, where this time derivative for the rear foot was greater or equal to zero, was defined as the start (t = 0 s), which signifies the first detectable movement. On average (32 maximal sprints) the rear foot lost contact with the floor at t= 0.152 ± 0.020 s.

Athletes ran a total of six 60-m sprints at respective efforts of 60%, 80%, 100%, 60%, 80%, and 100%. They started at their own initiative with a 5-min rest between sprints to provide enough time to recover. The runs at 60% and 80 % were used as an additional warm-up for the first 100% sprint and subsequently as active recovery from the first sprint. Only the two sprints at 100% were analyzed. The athletes were familiar with this manner of testing and with the equipment. 

## 3. Data Analysis

The IMU signals were analyzed using custom-written MATLAB (version 2018a, The Mathworks Inc., (Natick, MA, USA) scripts. This included scripts to automatically detect moments of touchdown and push-off of the feet during the 60-m sprints and the exact instances that the athletes contacted the mechanical timing gates. The procedure for step detection involved the gyroscope signal of the sensor’s x-axis that was orientated in the medial-lateral direction of the foot. First, the unfiltered signal was differentiated twice, then rectified, followed by low-pass filtering (second order Butterworth) at 10 Hz. The resulting signal displays pairs of positive peaks for each step, every first peak denoting the approximate instance of a touchdown, which is followed by a second peak denoting take-off. Identification of these peaks was done using the ‘findpeak’-function of Matlab. The exact moment of touchdown was defined as the instance where the twice differentiated and rectified gyroscope-x signal exceeded a threshold of 1°·s^−3^ in the 30 ms interval prior to the original (approximated) touch-down instance.

The onset of spikes in the unfiltered acceleration signals were used for the synchronization of the IMUs (see Test Procedures) and to determine the moments when the runners passed the mechanical timing rods. Each of the three acceleration signals was first differentiated and then rectified. Subsequently these signals were summed and the onset was defined as the instance that this summed signal exceeded a threshold of 20 m·s^−4^ for synchronization and 2 m·s^−4^ for mechanical timing.

Step duration was defined as the time between consecutive touchdowns. For the first step, the time elapsed between the first movement of the rear foot (t = 0 s) and the following touchdown served as step duration.

### 3.1. Video Analysis

The athletes were video recorded (30 Hz) from the side with ten cameras (Fujifilm XP60 Full-HD, (Fujifilm, Minato, Tokyo, Japan) mounted on tripods (1.2 m height) placed 8 m to the side of the sprint lane, from 2 to 38 m at 4-m intervals. To be able to correct for parallax during analysis, small cones were placed at exactly 1-m intervals on either side of the 1-m wide lane in which the athletes ran. This allowed the orientation of the perspective grid during analysis with free software (Kinovea 0.8.15 www.kinovea.org). The software was used to determine the distance from the start line at which the feet landed during all sprints. The tip of the spiked shoe was used for the manual digitization of all steps over the first 40 m. The tip of the shoe has zero velocity during ground contact. The fastest athlete in the group had the shortest ground contact times, which were about 100 ms at maximal speed. The video recorded at 30 Hz, thus the tip of the shoe remained still for at least three frames during ground contact, which was long enough for accurate digitization. Step length was defined as the step to step difference in distance. Stride length was defined as a complete left to right cycle and thus included two consecutive steps. 

Step velocity was calculated by the division of the video-derived step lengths by the step duration obtained from the IMUs on the feet. These video-derived step velocity-time relations were fitted (least squares) with a mono-exponential horizontal velocity-time function V_H_video_ (t) = V_Hmax_·(1 − e^−t/τ^) to investigate if, similar to what has extensively been shown for the athlete’s center of mass [3,19], the step velocity would also increase mono-exponentially over time (first hypothesis). In this function, V_Hmax_ is the athlete’s maximal sprinting velocity and τ a time constant, signifying how fast the athlete approaches V_Hmax_. The V_H_video_ (t) = V_Hmax_·(1 − e^−t/τ^) functions were extrapolated to 60-m. Similar to the procedure presented in Section 3.2., the fitted function was used to estimate the time at which the 60-m line was passed, which was compared with the measured (timing gate) crossing of the 60-m line. The moment that one of the feet crossed the 30-m line was determined with linear interpolation using the last touchdown before and the first touchdown after the 30-m line.

### 3.2. Timing Gate Analysis

To test our second hypothesis, in addition to the V_H_video_ (t) function, an alternative mono-exponential function was constructed. The alternative function was solely based on the 30-m and 60-m gate times: V_H_gates_ (t) = V_Hmax_·(1 − e^−t/τ^). For every sprint, the flying 30-m distance was divided by the difference between the measured 30-m and 60-m gate times (flying 30-m time) to obtain V_Hmax_. Subsequently, the time constant τ was optimized by computer iterations (0.0001 s increments). Iterations continued until the total error between the 30-m and 60-m times predicted from the V_H_gates_ (t)-function and the measured 30-m and 60-m times reached a minimum. This occurred as follows: With each iteration, the IMU-derived touchdown times of the steps were put into the V_H_gates_ (t)-function to obtain the step velocity at the moments of step touchdown. These step velocities were subsequently multiplied by the time differences between consecutive steps (step duration) to obtain step lengths. Then, the cumulative step length (distance run) was used to calculate the moments that the 30-m and 60-m lines would be passed with the current value of τ. Of course, it hardly ever occurred that the feet landed exactly at 30-m and 60-m. Therefore, the time and distance traveled from the last touchdown before 30-m and 60-m were first determined. Then, with linear interpolation between the last touchdown before and the first touchdown after the 30-m and 60-m lines, the time that one of the feet passed 30 m and 60 m was calculated. The iteration process continued until these calculated times matched the measured times at 30 m and 60 m as closely as possible (minimal difference).

## 4. Statistical Analysis

The statistical analysis was done using SPSS (version 23, IBM, Chicago, IL, USA). The data are presented as mean ± standard deviation (SD). Effects were considered significant if *p* < 0.05. Normal distribution of the data was confirmed with the Shapiro–Wilk test, visual inspection of the histograms, Q-Q plots, and box plots. To establish concurrent validity between the step and stride lengths estimated from the IMU data and the measured step and stride lengths obtained with the video reference, linear regression (reporting adjusted R^2^ and root mean square error, RMSE) and Bland–Altman (BA) analysis (bias ± limits of agreement (LOA)) was performed using the first 20 steps (covering about 35 m) of the two 60-m sprints of the sixteen participants, thus 20 × 2 × 16 = 640 steps were included, which amounted to 320 strides. 

## 5. Results

### 5.1. Video Reference

**Figure 1:**
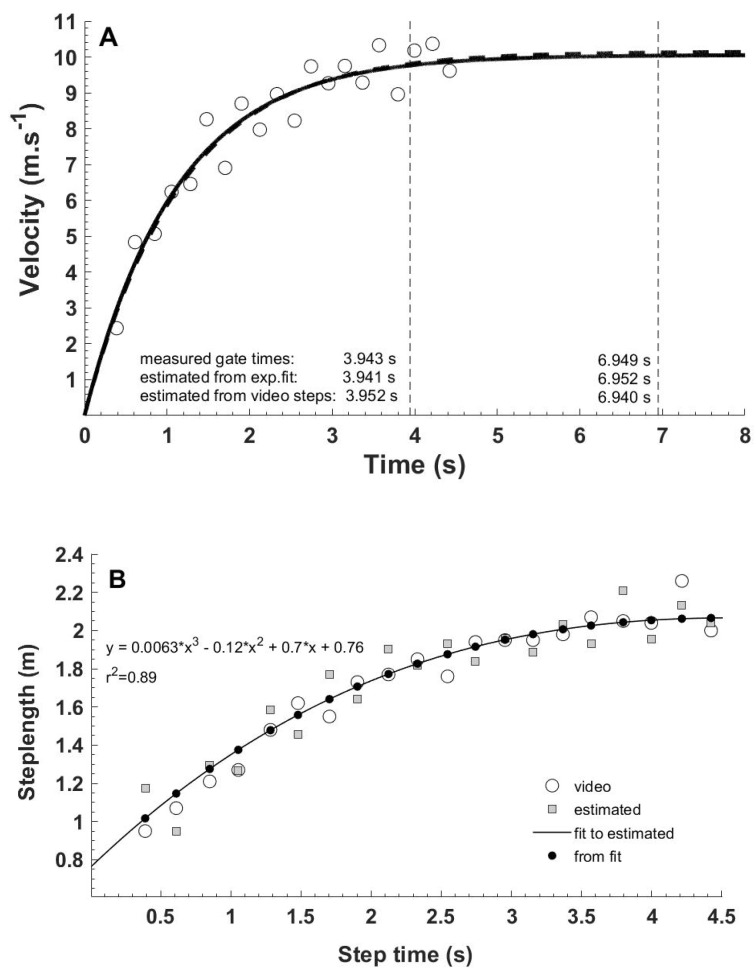
Velocity (**A**) and step length (**B**) during the 60-m sprint.

The thirty-two, video-based, step—velocity—time relations were very well described by mono-exponential functions (R^2^ = 0.99 ± 0.008). Data of all individual sprints are provided in the supplementary file. Figure 1A shows an example for the participant who had the lowest R^2^ (0.96). 

A typical example of a step—velocity—time function (open circles) derived from the reference step lengths (video) divided by IMU-derived step duration is shown in Figure 1A. These step-velocity data were fitted with the dashed mono-exponential function (R^2^ = 0.96). The continuous line depicts the mono-exponential function that was solely based upon the measured 30-m (3.943 s) and 60-m split times (6.949 s). Note that this function is very similar to, and almost completely covers, the reference function (dashed line). The vertical dashed lines indicate 30-m and 60-m passages of the feet. This athlete’s personal best was 10.85 s at the 100 m.

Figure 1B shows (same athlete) the relation between step time (touchdown times of the feet) for the first 20 steps of the 60-m sprint and estimated step length (grey squares). To obtain the estimated step length, step velocities derived from the timing gate-based mono-exponential function (solid line shown in Figure 1A) were multiplied by step duration measured with the IMUs. When the estimated step lengths (grey squares) were additionally smoothed by fitting a third order polynomial function (solid line in Figure 1B, R^2^ = 0.89), step length estimates (black dots) further improved and became even more similar to the video-reference values (open circles in Figure 1B). 

**Figure 2:**
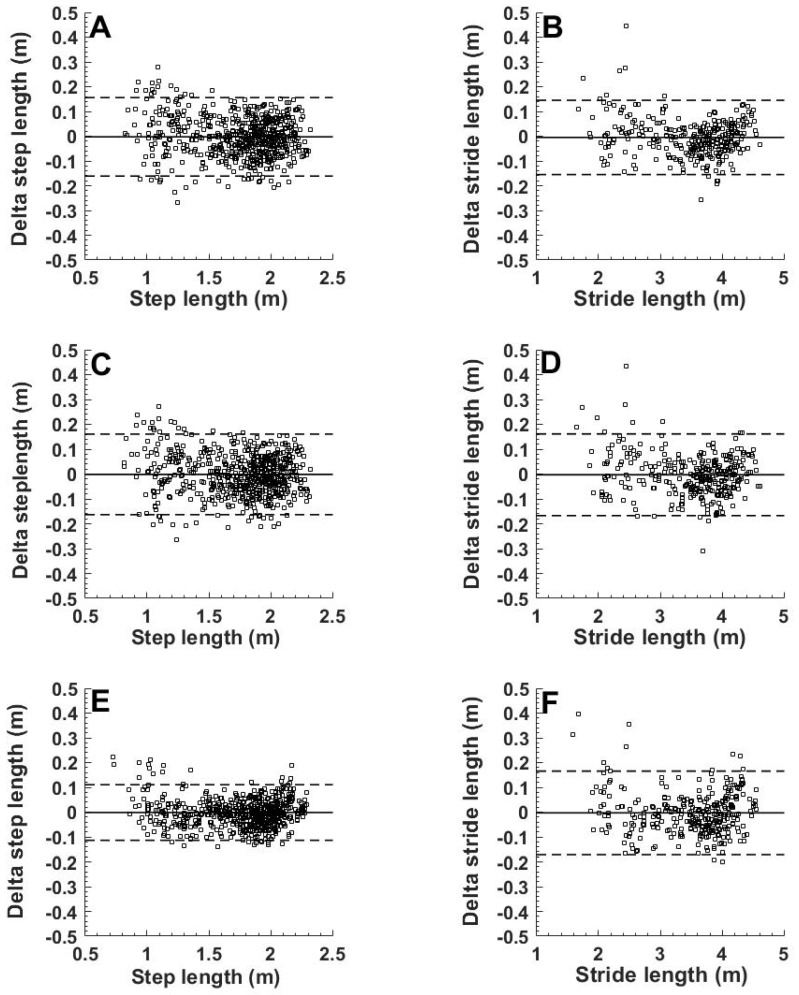
Bland—Altman plots for steps (**A**,**C**,**E**) and strides (**B**,**D**,**F**) with measured lengths from video as reference.

Bias (solid lines) and 95% limits of agreement (dashed lines) are depicted for: (1) Estimations derived with mono-exponential step velocity-time functions that were fitted (e.g. dashed exponential in Figure 1A) to the reference steps obtained with video (Figure 2A,B), (2) estimations derived with mono exponential step velocity-time functions (e.g. solid exponential in Figure 1A) fitted to the 30-m and 60-m gate times (Figure 2C,D), and (3) after an additional third order polynomial fit to those latter estimations (Figure 2E,F).

Using a mono-exponential step velocity-time function to estimate step-velocity, instead of using the actual step-velocities, introduces error in the step and stride length estimations. To illustrate the magnitude of these errors, estimated step and stride length were compared with the measured lengths (Figure 2A,B). As would be expected from the high proportion of explained variance presented above (R^2^ = 0.99 ± 0.008), estimated step lengths (n = 640) derived with these fitted exponential step-velocity-time functions showed a good linear relation (R^2^ = 0.95, RMSE = 0.079 m) with measured step lengths (BA: 0.002 ± 0.16 m, Figure 2A). Please note that for stride lengths, the linear correlation was higher (R^2^ = 0.99) while absolute errors (RMSE = 0.075 m) remained similar (BA: 0.005 ± 0.16 m, Figure 2B), implying 50% smaller relative errors.

### 5.2. 30-m and 60-m Sprint Times of the Feet Compared to Gate Timings

In the proposed method, gate times obtained at chest height are used to derive a mono-exponential velocity-time function of the feet. The time (obtained with interpolation) at which one of the feet crossed the 30-m line was strongly related to measured 30-m gate time (n = 32, y = 1.02x − 0.0477, R^2^ = 0.992, RMSE = 0.022 s). However, on average the feet were 0.045 s faster (bias 0.045 s, *p* < 0.05; LOA [0.088 0.0024 s]). Based on the extrapolated video-derived step velocity-time functions (Figure 1A), the time at which the feet were predicted to cross the 60-m was also highly related to the measured 60-m time (y = 1.03x + 0.181, R^2^ = 0.996, RMSE = 0.03 s), but it also was significantly faster (bias 0.048 s, *p* < 0.05; LOA [0.11–0.017s]). Thus, on average, the tip of one of the feet passed the 30-m line 0.045 s earlier than the chest. These results indicate that we should subtract 0.045 s from the gate times to use the gate times to obtain exponential velocity-time functions that accurately describe the velocity–time profile of the feet. Therefore, corrected gate times (= measured times minus 0.045 s) were used to derive the exponential velocity-time functions of the feet in the next paragraph.

### 5.3. Timing Gate Based Exponential Velocity-Time Functions

The predicted 30-m split time of the feet obtained with the timing gate based exponential velocity-time functions was 4.28 ± 0.25 s. It closely matched the corrected gate time at 30-m (R^2^ = 1.0, RMSE = 0.0015 s, bias (*p* < 0.05) 0.0024 s, LOA [−0.00073 0.0055]). Predicted 60-m time (7.59 ± 0.49 s) of the feet also closely matched the corrected 60-m gate time (R^2^ = 1, RMSE = 0.0016 s, bias (*p* < 0.05) −0.0025 s, LOA [−0.0056 0.00058]. Thus, fitting a mono-exponential velocity-time function solely based upon the measured 30-m (split) and 60-m times (−0.045 s) introduced systematic errors of only a few milliseconds. Thus, during maximal sprint acceleration, mono-exponential velocity-time functions constructed with the 30-m (split) and 60-m (finish) times as input accurately describe the increase of step velocity during individual sprint accelerations.

The step and stride lengths estimated from the timing gate-derived mono-exponential functions were strongly related (steps: R^2^ = 0.95, RMSE = 0.08 m, strides: R^2^ = 0.99, RMSE = 0.082 m) and similar to the measured video steps and strides (BA: −0.0015 ± 0.16 m; Figure 2C and −0.003 ± 0.16 m; Figure 2D). The linear relations and BA-plots are very similar to those derived from the exponential velocity-time fits of the measured step-velocities (Figure 2A,B). This also illustrates that using the timing-gate based step velocity functions (Figure 2C,D), instead of the functions obtained from all video-derived steps (Figure 2A,B), hardly increased the errors in estimated step and stride lengths.

The spread in Figure 2A,C is somewhat larger at lower step lengths that represent the shortest (first two) steps of the sprints. For five athletes, the length of the first step was overestimated considerably (15–22 cm) in both runs, while that of the second step was underestimated (e.g. Figure 1B) by a similar magnitude. These step-to-step errors largely canceled out in calculated stride length (Figure 2B,D). 

Step length prediction errors were substantially reduced when estimated step lengths (Figure 2C) were additionally smoothed by fitting a third-order polynomial function (e.g. Figure 1B) to obtain the final estimates. These final estimates were strongly related (R^2^ = 0.97, RMSE = 0.057 m) and similar to measured reference step-length (BA: −0.0016 ± 0.11 m; Figure 2E).

## 6. Discussion

Step velocities were calculated using video recorded step lengths and IMU-derived step times during maximal 60-m sprints. Similar to what others reported for the center of mass [3,19], the increase in step-velocity during acceleration followed a mono-exponential function in all individual sprints. Very similar mono-exponential velocity-time functions were constructed with only two timing gates placed at 30 m and 60 m. With these alternative functions and the IMU-derived step times, good estimates of stride and step length during maximal sprint acceleration were obtained, which is the most important new finding of the present study.

### 6.1. Mono-Exponential Velocity Increase

Horizontal velocity increases mono-exponentially during sprint acceleration ([3,19] and references therein), although some used a fourth-order polynomial to fit the velocity-time data (e.g. [20]). A mono-exponential function fitted our video-based step-velocity data very well (R^2^ = 0.99 ± 0.008). Based on the interpolation of these steps, the feet, on average, crossed the 30-m and 60-m lines 0.045 s earlier than the measured gate times. Thus, the tip of one of the feet crossed the 30-m and 60-m lines slightly ahead of the chest, while the chest usually contacted the mechanical timing rods. At the 30-m line, the average velocity was about 8.5 m·s^−1^ for the current athletes, which means that the tip of one of the feet was ~0.38 m (=8.5 m·s^−1^ × 0.045 s) ahead of the chest at ground contact. This distance is in line with the touchdown distance (about 0.32 m) reported for physical education students running at 8.04 m·s^−1^ [21]. There may have been some inter-individual differences, but gate times were corrected by a fixed value of 0.045 s for every athlete, as a straightforward approach to obtain the mono-exponential step-velocity time functions. 

Mono-exponential velocity time functions implicate that the athletes continued to increase their velocity slightly, even during the last part of the 60 m (e.g. Figure 1A). It can be questioned whether this occurred in reality. In world-class athletes, the decline in velocity only clearly occurs after 60 m [22,23], at the national level, sprinters reach their maximal velocity around 45 m [2] and non-specialists already around 30 m [3]. Our athletes were well-trained and probably continued to run at or close to maximal velocity between 40 and 60 m. The 30-m and 60-m times derived from the fitted mono-exponential step velocity-time functions (e.g. Figure 1A) agreed very well with the measured times. The fitting procedure caused a bias of a few milliseconds only. Together, our findings indicate that mono-exponential velocity-time functions provide good descriptions of the actual step velocity-time profile of the sprints in well-trained athletes.

To the best of our knowledge, our estimations of step length are considerably more accurate than previous predictions at high running speeds. Deriving accurate estimates of step length during maximal sprint acceleration from the IMU-signals using sensor fusion techniques [11,24] is not (yet) possible. During walking, stride length was predicted with reasonable accuracy (bias 1.5 cm and limits of agreement 13.3 cm) compared to an optical motion capture system [24]. The authors used an optimized fusion algorithm of gyroscope and acceleration signals coupled with de-drifted integration of the acceleration signals of IMUs placed on the feet. This method is challenging because noise will introduce drift which will amplify during integration. During walking, zero velocity updates can substantially reduce this drift, but during running, and even more during sprinting, this becomes problematic, as the sensors will never have zero velocity during maximal sprinting. In spite of these challenges, Brahms et al. recently reported estimates for stride length obtained with a foot mounted sensor that seem quite acceptable, with a bias of −3.2 cm and 15 cm limits of agreement [11]. However, in contrast to the present study, they had their participants run at constant speeds, thus ignoring the acceleration phase. Moreover, they only studied slow speeds (2.17–4.36 m·s^−1^) [11]. Zrenner et al. reported (their Figure 8a) stride length errors similar to those of Brahms et al. [11] and the present study. They included speeds from 2 to 6 m·s^−1^ in their analysis and showed that the error of stride length estimations increased above 5 m·s^−1^ for the different algorithms that they evaluated [12]. The athletes in the present study reached velocities higher than 5 m·s^−1^ already after two or three steps, and attained maximal velocities ranging from 8.0 to 10.5 m·s^−1^. Despite the inclusion of the acceleration phase and these high speeds, the errors in step length estimation were similar compared to the studies of Brahms et al. [11] and Zrenner et al. [12]. Therefore, we conclude that during maximal sprint acceleration, the present method to obtain stride length seems better than the existing IMU-based methods. The present method is attractive because it simply uses the IMUs to determine touchdown times of the feet, which in combination with the 30 m and 60 m times, provides reasonable estimations of step (and stride) length. The original step length estimations were subject to some error, but the step length estimations improved (RSME 5.7 cm) when the initially predicted step lengths were smoothed with a third order polynomial function (Figure 2C). This reduced relative RSME to about 5% for the first steps and even less for later steps. 

### 6.2. Limitations

The runners’ chest did not always contact first with the rods of our mechanical timing gates. Occasionally the arm or even the hand contacted the rod, which introduced some variation in the sprint times, but this also is the case with single beam optical timing gates.

The manual digitization of videos introduced a random error of a few cm in our reference step lengths. Unfortunately, we did not have 40 m of a more accurate optical measurement system at our disposal that others used to obtain step length [2,7].

Our unsmoothed estimates of step length were subject to some error (RSME 8.2 cm, Figure 2A). Particularly during the first two steps, which usually are only about 1 m, the error was relatively large (about 8 %). Differences in placement of the feet in the anterior-posterior direction of only a few centimeters relatively to the vertical projection of body’s center of mass, possibly are relevant for performance [8,25]. Consequently, our step length predictions do not seem accurate enough for very specific analysis of individual foot placements during single sprints. However, the method is simple and it is easy to repeat measurements. This opens the possibility to average the athlete’s steps from several sprints, which will minimize random error, including occasional missteps of the athletes. Therefore, the proposed method, including the additional polynomial filtering of the initially estimated step lengths, probably is accurate enough to compare step lengths between athletes of different skill levels and to evaluate (training-induced) changes in individual sprint acceleration technique. 

In relation to the previous point, our method also is not sensitive enough to accurately quantify the natural variations in step length, neither within a single sprint nor between sprints of the same athlete. This shortcoming probably relates to the assumption of a perfect mono-exponential increase in step velocity. In reality, there is some natural variation from step to step (e.g. Figure 1 and Figure 2C), which may partly be due to imperfect execution of the acceleration by the athlete [26]. However, when two consecutive steps were combined (step one with step two, step three with step four, etc.) to obtain stride length, the relative error was halved to reach maximum values of about 4% (RMSE), which were obtained for the shortest (2 m) strides (Figure 2B). To some extent this improvement may be the result of averaging out measurement errors of consecutive steps. In addition, consecutive touchdowns probably are not independent events. When an athlete slightly oversteps, the next step probably is a bit shorter to compensate. 

A final limitation is that, with the presented method, only maximal sprints executed in a straight line can be analyzed. However, maximal sprint capacity is important in many sports and maximal straight-line (30–60 m) sprints are commonly used to evaluate sprint performance in many sports. 

### 6.3. Practical Considerations

In principle, the velocity-time function of sprinters can also be derived with a series of more than two (optical) timing gates or directly with radar or laser equipment. Although the latter devices measure instantaneous velocity rather well, the data need filtering and the accuracy depends on properly aiming these devices at the sprinters lower back [27]. In practice, this is not always easy. Moreover, the velocity of the lower back and the velocity of the steps may be slightly different. It remains to be investigated whether it also is possible to obtain step length when the velocity recorded with radar or laser is combined with IMU-derived touchdown times of the feet.

The athletes in the present study did not use starting blocks, because block-starts require a technique that was not mastered to the same extent by all the athletes. Football, rugby, and soccer players also do not use starting blocks, which facilitates the generalization of the present results to other sports. An additional strength of the present study is that the performance of the present athletes covered quite a broad range, although it unfortunately only included three women. Thus, our results are representative of athletes with different sprint capacities. The foregoing considerations suggest that the proposed method of step length estimation most likely applies to sportsmen and women in many other sports.

Speed is the product of step rate and step length. IMUs attached to the shoes of athletes provide valuable information about ground contact time, airtime and step rate, which together with step length are key performance determining variables in sprinting [1,2,3,5,6,7]. For clarity, we only presented step and stride length and how these can be derived in a simple, cost-effective manner. Of course, step length can also be obtained directly from video, as was done to get the reference values in the present study, but this is time-consuming and impractical. Alternatively, there are optical measurements systems integrated into bars placed on both sides along the running lane [2,7], that measure step length with good reliability [2]. However, these systems are heavy, quite expensive and not easy to use on a day-to-day basis at different locations. In contrast, IMUs are inexpensive, small, light-weight, and easy to use. Moreover, IMUs detect the first movement at the start, and therefore it is possible to obtain very clean sprint times when the sensors are appropriately synchronized with timing gates. It is also possible to include the reaction time in the sprint time measurements. To do this, an extra IMU has to be attached to a clapper board, the sound of which serves as a starting signal for the athletes. A final important consideration is that, in our experience, athletes have no problems wearing the IMUs and even run with them during competition. 

In conclusion, 30-m and 60-m times in combination with IMU-derived step timing suffice to obtain good estimates of stride and step length during maximal sprint acceleration. Step length estimates complement other important sprint performance related variables (step rate, ground contact, and airtime) that can be accurately measured with IMUs and are used by coaches to monitor the training process and technical development of their athletes. 

## Supplementary Materials

The following are available online at https://www.mdpi.com/2075-4663/7/9/202/s1, individual step data: Supplementary material. XLSX
